# Food insecurity impacts healthy lifestyle practices and medication adherence among persons with hypertension in Colombia and Jamaica: Findings from a cross-sectional study

**DOI:** 10.21203/rs.3.rs-8135598/v1

**Published:** 2025-12-09

**Authors:** Nadia R. Bennett, Jacqueline P. Duncan, Siyi Geng, Katherine T. Mills, Paul K. Whelton, Althea Bailey, Amanda Anderson, Meryl Cusimano, Paola Lanza, Patricio Lopez-Jaramillo, Trevor S. Ferguson, Marshall K. Tulloch-Reid

**Affiliations:** The University of the West Indies, Mona; The University of the West Indies, Mona; University of Texas Southwestern Medical Center, Peter O’Donnell Jr. School of Public Health; Tulane University Celia Scott Weatherhead School of Public Health and Tropical Medicine; Tulane University Celia Scott Weatherhead School of Public Health and Tropical Medicine; The University of the West Indies, Mona; University of Alabama at Birmingham; Tulane University Celia Scott Weatherhead School of Public Health and Tropical Medicine; University of Texas Southwestern Medical Center, Peter O’Donnell Jr. School of Public Health; Universidad de Santander (UDES); The University of the West Indies, Mona; The University of the West Indies, Mona

**Keywords:** socioeconomic status, food insecurity, hypertension, medication adherence, lifestyle, LatinAmerica and the Caribbean

## Abstract

**Background:**

Adherence to medications and healthy lifestyles are important health behaviours for hypertension control and may be influenced by food insecurity and socioeconomic status (SES). We examined associations between food insecurity and SES with medication adherence and healthy lifestyle practices among patients with hypertension in Colombia and Jamaica.

**Methods:**

In a cross-sectional survey of hypertensive patients attending primary health centres in Colombia and Jamaica we collected self-reported demographic, education and employment information. Food insecurity was measured using two questions from the modified United States Department of Agriculture (USDA) food security instrument – was there sufficient money for 1) weekly food purchases and 2) healthy foods. Medication adherence was classified as high vs. low/medium using the IMPACTS-MAS questionnaire. Healthy lifestyle was scored using a 6-point scale as unfavourable (score ≤ 3) or favourable (score 4–6), 1 point each for eating less salt, exercising regularly, consuming ≥ 2 servings fruits and ≥ 3 servings vegetables daily, and reduced alcohol consumption or 2 points for abstinence. Multivariable logistic regression with medication adherence categories and lifestyle practice scores as dependent variables assessed associations with food insecurity and SES.

**Results:**

Of 576 participants (Colombia 288; Jamaica 288), Colombians were older (66.5 years vs. 62.5 years, p < 0.001), had higher education attainment and were less likely to be employed/retired. Colombians had less food insecurity (64.6% vs. 89.0% p < 0.001), higher medication adherence (88.2% vs 50.7% p < 0.001) and more favourable lifestyle practices (86.2% vs. 47.2% p < 0.001). Food insecurity in both countries was inversely related to education, having private health insurance, high medication adherence and healthy lifestyle practices. In multivariable models, food insecurity was associated with increased odds of unfavourable lifestyle practice scores, (OR = 2.4; 95% CI: 1.1, 5.2 p = 0.028) and poor medication adherence (OR = 1.9; 95% CI: (1.0, 3.7) p = 0.072), after adjusting for age, sex, country, marital status, education and employment and having ≥ 2 chronic illnesses. No associations with the other SES examined.

**Conclusion:**

Food insecure hypertensive patients had increased odds of unfavourable lifestyle practices and were twice as likely to have poor medication adherence. Strategies to address food insecurity may have a positive impact on hypertension control in low resource settings.

## Background

Cardiovascular diseases (CVDs) are the most common cause of non-communicable disease (NCD) deaths, accounting for 19 million deaths annually [1]. Hypertension, defined as an average systolic blood pressure ≥ 140 mm Hg, diastolic blood pressure ≥ 90 mm Hg, or taking antihypertensive medication, affects approximately 1.4 billion people worldwide and is the most important modifiable risk factor for CVD and disability-adjusted life years (DALYs) [2, 3].

Healthy lifestyle practices – consuming less salt, eating more fruits and vegetables, decreased or no consumption of alcohol, and regular exercise – and use of antihypertensive medications has been shown to substantially reduce blood pressure, reduce major adverse cardiovascular events, cardiovascular mortality, healthcare costs, and improve quality of life [4–6]. The World Health Organisation’s (WHO) Global Hearts initiative and the American Heart Association’s (AHA) “Life’s Essential 8” program recognizes the importance of healthy lifestyle choices on cardiovascular health and promote them as an important means for primary and secondary prevention of cardiovascular disease [7, 8].

Poor adherence with pharmacological treatment is an important reason for uncontrolled hypertension [9, 10]. In one study, approximately 50% of patients discontinued prescribed antihypertensive medications within one year of initiating treatment [11]. The WHO describes non-adherence to pharmacological treatment as a problem of striking magnitude [12]. Factors such as socioeconomic status, the use of complex medication regimens, multimorbidity, and poor-quality of physician-patient relationships have been associated with poor adherence to pharmacological treatment, particularly in low and middle income countries (LMICs) with limited access to health resources [13, 14].

Food insecurity, defined as the inability to access adequate and nutritious food for an active and healthy life due to factors such as financial constraints and food unavailability [15], has been associated with unhealthy lifestyle practices and poor medication adherence in North America and Europe [16, 17]. In 2022, the Food and Agriculture Organisation (FAO) of the United Nations reported a 29.6% prevalence of moderate and severe food insecurity globally. However, higher levels of moderate and severe food insecurity were reported for Latin America (Mesoamerica 34.5%, South America 36.4%) and the Caribbean (60.6%) [18], compared to North America and Europe (7.9%) [19]. During the 2019 coronavirus disease (COVID-19) pandemic, secondary analysis of a survey of over 1 million adults in LAC estimated that the prevalence of food insecurity was approximately 76% [20].

Previous studies suggest that individuals with food insecurity take medications less frequently than prescribed, due to costs, and payments for medications are often deferred to save money to buy food [16]. This behaviour can be taken within the wider context of the social determinants of health (SDOH) and includes access to education, stable employment and financial resources. Low educational attainment and unemployment often exacerbate food insecurity and hinders adherence to antihypertensive medications and healthy lifestyle choices [21, 22]. LAC countries with a high prevalence of uncontrolled hypertension combined with high levels of food insecurity and unemployment are especially vulnerable to increased CVD morbidity. Despite the compelling implications of these data, few studies have been conducted in the region to examine the association between food insecurity and adherence to antihypertensive medications and healthy lifestyle practices.

The current study was conducted as part of a needs assessment investigation in preparation for the Caribbean and South America Team-based Strategy to Control Hypertension (CATCH) Study. The CATCH Study is a National Institutes of Health (NIH) supported cluster randomized trial to test the implementation and effectiveness outcomes of implementing and scaling up a team-based care strategy for blood pressure (BP) control in Colombia and Jamaica. In this paper we examine associations between socioeconomic status, food insecurity and adherence to healthy lifestyle behaviours and antihypertensive medications among patients with hypertension in both settings.

## Methods

### Participants and Study Procedures

The design of this cross-sectional study has been previously described [23]. Between August 2021 and February 2022, 576 patients with hypertension were enrolled and completed an interviewer-administered questionnaire in Colombia and Jamaica (See Supplement 1 for the full questionnaire). In Colombia, patients with hypertension were randomly selected from four primary care clinics using an electronic medical records database. In Jamaica, consecutive patients with hypertension from 10 primary care clinics were selected on the days they visited the clinic. Due to the COVID-19 pandemic, all interviews were conducted by trained research assistants by phone.

#### Measurements

The survey gathered information on demographic characteristics (sex, age, education, employment, and marital status), geographic location (country of residence and urban/rural setting), and health status (comorbidities, use of antihypertensive medications, age at hypertension diagnosis). Data were also collected on health care financing, food insecurity, adherence to antihypertensive medications, lifestyle factors, and the impact of COVID-19 on their health and care. Multimorbidity was defined as the co-occurrence of ≥ 2 self-reported chronic conditions (diabetes, high cholesterol, depression, anxiety, kidney disease, stroke, heart attack/heart disease, overweight/obesity) [24]. Health care financing was categorized as follows: Insurance (Insurance by employer or private insurance purchased by participant or spouse), self-financed (out-of-pocket or family/friends), government (government subsidy or program) and charity.

#### Socioeconomic status

Socioeconomic status was measured using education attainment and employment status. To classify education participants were asked *“What is your highest level of education”* and patients classified as having less than high school, high school and more than high school education. For employment status participants were asked “Are you currently working including self-employment”. There were then classified into two categories – employed/retired vs. other (which included students, housewives, unemployed and disabled persons).

#### Food insecurity

Food insecurity was assessed by two questions from the USDA food security instrument: *“The food that we bought just didn’t last, and we didn’t have money to get more”* and *“We couldn’t afford to eat healthy meals with more fruits and vegetables”* [25]. Persons were classified as food insecure if they responded “true most of the time” or “true some of the time” to either of these two questions.

#### Lifestyle and Medication Adherence

Lifestyle adherence was scored on a 6-point scale where 1 point was assigned for each of the following: currently eating less salt, exercising regularly, eating adequate amounts of fruit (at least 2 servings daily), vegetables (at least 3 servings daily). Points were assigned for alcohol moderation with reduction of intake receiving one point and complete abstinence from alcoholic beverages receiving 2 points. Lifestyle adherence was grouped as unfavourable (score ≤ 3) or favourable (score 4–6).

Medication adherence was based on the Implementation of Multifaceted Patient-Centred Treatment Strategies for Intensive Blood Pressure Control medication adherence scoring system (IMPACTS-MAS) which used 2 questions: “*Over the last 7 days, on how many days did you not take any blood pressure pills?*” where points ranged between 0 (if no medications were taken for 7 days) and 4 (if all medications were taken for 7 days) and “*Over the last 7 days, on how many days did you cut back (or not take the full dose) of your blood pressure pills?*” where points ranged between 0 (if no medications were taken for 7 days) and 2 (if all medications were taken for 7 days). The sum of the responses to both questions were calculated and patients were classified as having high (6 points), medium (5 or 5.5 points) and low (< 5 points) medication adherence [26].

### COVID-19 impact

Given that the survey was conducted during the COVID-19 pandemic, we also sought to assess the impact of the pandemic restrictions on healthcare access. COVID-19 impact was determined based on responses to the question: *“Were you not able to see a doctor or get prescription medications due to any of the following reasons – a) pharmacy, hospital or clinics closed; b) less time to see a doctor or get medications due to curfew restrictions; c) fear of contracting coronavirus if you leave the house”*. After assigning “1” point to each “yes” response, a total score was used to categorize COVID-19 impact as minimal (score = 0), moderate (score = 1 to 2), and substantial COVID-19 impact (score = 3).

#### Sample Size

Using PASS 15 (NCSS, LLC) software based on assumption of stratification by sex and urban/rural status, a minimum sample of 288 participants per country was calculated to detect a 20% response proportion with a 95% confidence interval from 10%−30%.

### Statistical Analysis

Analyses were done using SAS 9.4 (SAS Institute, Cary, NC). Frequency tables with percentages were generated and descriptive analyses used for the baseline population by food insecurity status. Mean and standard deviation were reported for continuous variables. Data were weighted based on distribution of age groups, sex, and residential areas within the respective target population of each country as assessed from clinic registers. In analyses that included participants from both countries, combined weights were applied. Sample sizes and frequency numbers are unweighted but means standard deviations (SDs) and percentages are weighted. Wald Chi-square test (or Fisher’s exact test for sparse data) was performed for differences between categorical variables, and Student’s t-test was used for differences between continuous variables, comparing Colombia and Jamaica.

Bivariate analyses were done to explore associations between socioeconomic status and food insecurity with variables of interest including medication adherence and lifestyle practice score. The outcomes were dichotomized – medication adherence high vs. medium/low and lifestyle adherence – favourable vs. unfavourable. Using a p-value level of ≤ 0.2 from the bivariate models, logistic regression was used to build the final multivariable model. For medication adherence, we adjusted for age, sex (male vs. female), country, marital status (married/common law vs. not married/common law) and health care financing (having private health insurance vs not). For favourable lifestyle, we adjusted for age, sex (male vs. female), marital status (married/common law vs not married/common law), multimorbidity (having 2 or more vs. 0–1 chronic illnesses) and COVID-19 impact (minimal vs moderate and substantial).

## Results

There were 576 participants (288 Colombia, 288 Jamaica) in this study, of which 31.5% were male. The Colombian participants were older than their counterparts from Jamaica (mean age 66.5 years vs. 62.5 years, p < 0.001) and were more likely to be in a married/common law relationship (58.9% vs. 40.6%, p < 0.001). Higher education levels were observed in Colombia, where more participants achieved “more than high school” education (30.3% vs. 17.2%, p = 0.01), while Jamaicans had higher employment/retiree levels (76.1% vs. 64.0%, p = 0.01) compared to Colombians (Supplement 2: Table 1). The Colombian participants had a shorter mean duration of hypertension compared to their counterparts in Jamaica (11.1 years vs. 16.5 years, p < 0.001) and had a lower percentage with multimorbidity though the difference was not statistically significant (39.2% vs. 49.5%, p = 0.05). In Colombia, private insurance was the main source of health care financing (88.3%) while government subsidy, meaning government, self, family friends and or charity was the primary source in Jamaica 92.4%), p < 0.001. Colombians had lower levels of food insecurity (64.6% vs. 89.0% p < 0.001) but greater proportion with higher medication adherence scores (88.2% vs 50.7% p < 0.001) and favourable lifestyle adherence 86.2% vs. 47.2% p < 0.001). (Supplement 2).

### Food Insecurity

Table 1 describes the characteristics of participants of the study stratified by level of food insecurity. Overall, 440 (76.1%) of the participants were food insecure where the mean age and sex distribution of those who were food insecure compared to those who were food secure was similar. As education level increased food insecurity prevalence decreased (less than high school 42.2%, high school 39.4%, more than high school 18.3%, p < 0.001). Food insecure compared to food secure participants were also more likely to have no private insurance (58.5% vs. 21.3% P < 0.001), low/medium medication adherence scores (34.1% vs. 14.3% p < 0.001) and unfavourable adherence to favourable lifestyle practices (38.3% vs. 12.4% p < 0.001).

### Socioeconomic Status

Table 2 shows food insecurity, lifestyle practices and self-reported medication adherence stratified by educational attainment. We found that higher education was associated with less food insecurity. For example, 84.7% of those who had less than high school education was food insecure compared to 57.8% of those who had more than high school education (p < 0.001). However, medication adherence and lifestyle adherence scores were not significantly associated with education levels. We found that 28.5% participants with less that high school education compared to 27.7% with more than high school education had low/medium adherence (p = 0.817) and 34.8% participants with less that high school education compared to 22.7% with more than high school education had an unfavourable lifestyle adherence score (p = 0.067). (Supplement 2: Table 2: Baseline Characteristics Stratified by Education Level)

Table 3 shows the characteristics of participants of the study stratified by employment status. We found no significant associations between employment status and food insecurity, medication adherence and lifestyle adherence. (Supplement 2: Table 3: Baseline Characteristics Stratified by Employment Status)

Table 4 shows the associations between education, employment and food insecurity and unfavourable lifestyle adherence. In unadjusted models, those with a high school education had almost two times the odds of unfavourable lifestyle scores compared to those with more than a high school education (OR: 1.9, 95%CI:1.0, 3.4, p = 0.041). Participants who were food insecure had 4 times the odds of having unfavourable lifestyle adherence (OR: 4.4, 95% CI: 2.2, 9.0, p < 0.001). This remained significant in the fully adjusted model that included age, sex, country, marital status, multi-morbidity and COVID-19 impact (OR: 2.4, 95% CI:1.1, 5.2, p = 0.028).

Table 5 shows results for the associations between education, employment and food insecurity and antihypertensive medication adherence. In the unadjusted model, those who were food insecure had 3 times the odds of poor medication adherence compared to those that were food secure (OR: 3.1, 95% CI:1.7, 5.6, p < 0.001). This effect was attenuated after adjusting for age, sex, country, marital status, multimorbidity and COVID impact (Adjusted OR: 1.9, 95% CI: 1.0, 3.7, p = 0.072). There were no associations between education or employment and medication adherence in adjusted and unadjusted models.

## Discussion

In this study of patients with hypertension who were being treated at primary care clinics in Colombia and Jamaica, high levels of food insecurity were recorded in both countries. Food insecurity but not socioeconomic status was associated with poor adherence to recommended lifestyle practices and poor medication adherence, although the association with medication adherence was attenuated and no longer significant after adjustment for potential confounding variables. This association between food insecurity and favourable lifestyle was not fully explained by other socioeconomic factors or the country in which participants lived.

Latin America and the Caribbean has achieved the millennium development goal target of reducing the number of people suffering from hunger [27]. However, the COVID-19 pandemic, climate change and natural disasters, global political instability and increases in the cost of living have led to increasing food insecurity globally [27]. In 2022 FAO reported that the levels of food insecurity in LAC were 37.5% compared to the world estimate of 29.6%. The prevalence of moderate or severe food insecurity in South America was 37.5% and 60.6% in the Caribbean [18].

The prevalence of food insecurity in Colombia reported in this study (65%) was similar to the 66% noted in a previous national report [28]. However, in Jamaica we found a higher level of food insecurity (89%) than was previously reported (25–75%) [29, 30]. These differences may be attributed to the study population (older persons with hypertension), the composite score used and the timing of the study. We did not identify studies that explored food insecurity in patients with hypertension from LAC. It is possible that the costs of managing blood pressure would pose an additional strain and contribute to the higher prevalence of food insecurity that we observed [31]. In addition, the study was conducted during the COVID-19 pandemic. Studies confirm that all four food insecurity dimensions (availability, accessibility, usability and sustainability) were compromised resulting in substantial price increases of healthy foods during the pandemic [32].

In our survey, food insecurity was based on 2 of the 6 items in the six-item short form of the USDA food security instrument: the ability to purchase sufficient food or purchase healthy foods in our study [25]. Other elements of food insecurity in the USDA food security instrument include cutting meal size and intake, feeling hungry but not being able to eat, and the frequency with which these disruptions occur. It is possible that variations in food security in surveys are due to variations in the data collection tools. Nevertheless, food insecurity is consistently high reinforcing the urgent need for effective strategies.

We found a possible association between food insecurity and medication adherence, which is in keeping with the published literature [33, 34]. The methods for recognizing medication adherence, and the instruments used to measure adherence in other studies have varied widely. We used the IMPACTS-MAS scoring system [26] which was not used in the previous studies conducted in Jamaica or Colombia. A 2021 meta-analysis showed that food insecurity decreased the odds of medication adherence by 44% [34]. Like our findings, other employment status (i.e. unemployed, housewife, student, disabled)and less education did not have a significant association with medication adherence. Cost-related underuse and medication restricting behaviours are some issues that have been identified in other studies examining the relationship between food insecurity and adherence [35, 36].

Few studies report on lifestyle adherence in LAC, and the evaluation of lifestyle adherence is a complex construct. There are different combinations of measures when lifestyle adherence is reported in the literature. It is most frequently reported by a combination of dietary adherence (eating less salt), physical activity, medication adherence, weight management, smoking cessation and alcohol moderation. The lifestyle adherence score created for this study was comprehensive and took into account the individual components (eating less salt, exercising regularly, decreasing alcohol consumption or didn’t drink, eating adequate (at least 2 servings daily) fruits and adequate (at least 3 servings daily) vegetables) of the score.

We found that food insecurity decreased the odds of favourable lifestyle. Other studies have shown that food insecurity is inversely associated with favourable lifestyle factors including physical activity, diet, alcohol use and sleep quality[37–39]. For example, Kehoe et al showed that food insecurity affected the diet quality of young women in South Africa [40]. Alcohol abuse/dependence was associated with an increased odds of food insecurity among homeless men and women in the USA ((OR = 2.72, CI._95_ = 1.55, 4.77) [39]. For physical activity, authors hypothesize that external factors such as employment and long work hours of low-income food-insecure households may contribute to less physical activity [41]. Similarly, violence in communities may restrict physical activity for food insecure families. Of note, both Jamaica and Colombia have high homicide rates. Further studies in Jamaica and Colombia are needed to better understand the complex relationship between food insecurity and maintaining a healthy lifestyle.

### Strengths and Limitations

This study adds to the body of literature on lifestyle practices and determinants of BP control in two different countries in LAC, which has a high burden of CVD. The recruitment of participants from two countries with different health systems and cultural practices provides information on the possible role of food insecurity in BP control and possible targets for programmes to reduce the burden of hypertension.

One limitation is that other measures of SES and SDOH were not included in this study. Additionally, we relied on self-reported adherence to medication and healthy lifestyle, which may have resulted in reporting bias and misclassification of the outcomes. Information bias and misclassification could also have occurred due to the composite scores used for classification of lifestyle practices, medication adherence and food insecurity. The lifestyle adherence score was created for this study and, although validated in other populations, the instrument used to assess medication adherence (IMPACTS-MAS) [26] has not been previously used in Colombia or Jamaica. For food insecurity, only the first two (of six) questions of the modified USDA food security instrument were used. Structural and cultural factors such as policies and cultural practices are possible confounders or effect modifiers that were not captured in this study. Additionally, data collection occurred during the COVID-19 pandemic which had an impact on both food insecurity and lifestyle adherence with impacts differing between countries [20, 42]. Therefore, these findings may not reflect the usual practices.

Finally, it would have been useful to determine levels of food security (mild, moderate and severe) and their impact on medication adherence and favourable lifestyle. This may be explored in future studies.

## Conclusion

Persons with hypertension who were food insecure had an increased odds of unfavourable lifestyle practices and were twice as likely to have poor medication adherence. This study provides valuable insight into the complex interplay between socioeconomic factors including food insecurity and hypertension control in Colombia and Jamaica. Consistently taking medications and making healthy lifestyle choices are necessary to maintain good health and slow progression of chronic diseases. Given the high prevalence of food insecurity and its role in lifestyle and medication adherence, our findings suggest that public health policies, programmes and multi-sectoral strategies are urgently needed if we are to effectively prevent and control hypertension in LAC.

## Supplementary Material

Supplementary Files

This is a list of supplementary files associated with this preprint. Click to download.

- FIandHTNBMCPubhlthSupplementSurveySubmission.pdf
- FIandHTNBMCPubhlthSupplementSubmissionupdated.docx

## Figures and Tables

**Table 1:** Characteristics of Study Participants by Food Insecurity Status*

Characteristics	Food Insecure N = 440	Food Secure N = 136	P-value for group difference
**Demographic Characteristics**			
**Age, Mean (SD)**	64.8 (11.4)	64.5 (11.4)	0.845
**Sex, n (%)**			0.663
Male	198 (30.9)	76 (33.2)	
Female	242 (69.1)	60 (66.8)	
**Country, n (%)**			<0.001
Colombia	180 (45.1)	108 (78.4)	
Jamaica	260 (54.9)	28 (21.6)	
**Location, n (%)**			0.040
Urban	211 (65.6)	79 (76.0)	
Rural	229 (34.4)	57 (24.0)	
**Education Level, n (%)**			<0.001
Less than High school	185 (42.2)	30 (24.2)	
High School	182 (39.4)	45 (33.3)	
More than High school	73 (18.3)	61 (42.5)	
**Employment status, n (%)**			0.176
Employed/Retiree	298 (67.7)	113 (76.1)	
Other**	142 (32.3)	23 (23.9)	
**Multimorbidity, n (%)**			0.093
Yes (≥ 2 conditions)	211 (46.7)	58 (35.9)	
No	229 (53.3)	78 (64.1)	
**Healthcare financing, n (%)**			<0.001
Private Insurance	158 (41.5)	108 (78.7)	
No private insurance***	282 (58.5)	28 (21.3)	
**Medication adherence Score, n (%)**			<0.001
Low/medium	156 (34.1)	23 (14.3)	
High	276 (65.9)	109 (85.7)	
**Lifestyle Factors, n (%)**			
Eat less salt	423 (96.5)	131 (97.8)	0.389
Exercising regularly	216 (44.3)	97 (68.5)	<0.001
Decrease/don’t consume alcohol	435 (98.6)	134 (98.4)	> 0.999†
Eat ≥ 2 servings of fruits	177 (44.3)	87 (62.7)	0.006
Eat ≥ 3 servings of vegetables	151 (36.5)	93 (65.0)	<0.001
**Lifestyle adherence, n (%)**			<0.001
Unfavourable (≤ 3)	174 (38.3)	22 (12.4)	
Favourable (4–6)	266 (61.7)	114 (87.6)	
**Covid −19 Impact, n (%)**			<0.001
Minimal	291 (65.2)	44 (28.5)	
Moderate/Substantial	149 (34.8)	92 (71.5)	

* The sample sizes and actual frequency numbers are unweighted but means (SDs) or percentages are weighted.

** unemployed, housewife, students, disabled

*** no private insurance- meaning government, self, family friends and charity

† Fisher’s exact test

**Table 2:** Food insecurity, Medication and Lifestyle Adherence of Study Participants by Socioeconomic Status as measured by Educational Attainment*

Characteristics	Less than High School (N = 215)	High School (N = 227)	More than High School (N = 134)	P-value for Group Difference
**Food Insecurity, n (%)**				<0.001
Yes (Insecurity)	185 (84.7)	182 (79.0)	73 (57.8)	
No (Security)	30 (15.3)	45 (21.0)	61 (42.2)	
**Medication Adherence Score, n (%)**				0.817
Low/medium	62 (28.5)	78 (31.1)	39 (27.7)	
High	148 (71.5)	146 (68.9)	91 (72.3)	
**Lifestyle Factors, n (%)**				
Eat less salt	209 (96.1)	215 (96.6)	130 (98.1)	0.577†
Exercising regularly	120 (49.5)	111 (44.7)	82 (59.3)	0.114
Decrease/don’t consume alcohol	212 (97.8)	225 (99.1)	132 (98.8)	0.533†
Eat ≥ 2 servings of fruits	91 (44.4)	98 (48.8)	75 (55.5)	0.286
Eat at ≥ 3 servings of vegetables	80 (39.6)	91 (41.0)	73 (53.0)	0.125
**Lifestyle adherence, n (%)**				0.067
Unfavourable (≤ 3)	72 (34.8)	88 (35.5)	36 (22.7)	
Favourable (4–6)	143 (65.2)	139 (64.5)	98 (77.3)	

* The sample sizes and actual frequency numbers are unweighted but means (SDs) or percentages are weighted.

† Fisher’s exact test

**Table 3:** Food Insecurity, Medication and Lifestyle Adherence of Study Participants by Employment Status*

Characteristics	Employed/Retiree (N = 411)	Other** (N = 165)	P-value for Group Difference
**Food Insecurity, n (%)**			0.176
Yes (Insecurity)	298 (73.9)	142 (81.1)	
No (Security)	113 (26.1)	23 (18.9)	
**Medication Adherence Score, n (%)**			0.088
Low/medium	138 (31.9)	41 (23.4)	
High	265 (68.1)	120 (76.6)	
**Lifestyle Factors, n (%)**			
Eat less salt	394 (96.6)	160 (97.2)	0.743
Exercising regularly	237 (52.6)	76 (44.2)	0.163
Decrease/don’t consume alcohol	406 (98.4)	163 (98.8)	>0.999†
Eat ≥ 2 servings of fruits	191 (47.6)	73 (51.3)	0.545
Eat ≥ 3 servings of vegetables	166 (37.9)	78 (56.0)	0.003
**Lifestyle adherence, n (%)**			0.097
Unfavourable (≤ 3)	145 (34.7)	51 (26.2)	
Favourable (4–6)	266 (65.3)	114 (73.8)	

* The sample sizes and actual frequency numbers are unweighted but means (SDs) or percentages are weighted.

** unemployed, housewife, students, disabled

† Fisher’s exact test

**Table 4:** Crude and Adjusted odds Ratios exploring the Association between Education, Employment, Food Insecurity and Unfavorable Lifestyle*#

	Crude Odds Ratio (95% CI)	P-value	Adjusted Odds Ratio* (95% CI)	P-value
**Education**				
More than high school	Reference		Reference	
High school	1.9 (1.0, 3.4)	0.041	1.4 (0.8, 2.7)	0.258
Less than high school	1.8 (1.0, 3.3)	0.052	1.4 (0.7, 2.7)	0.366
**Employment**				
Employed/Retired	Reference		Reference	
Other **	0.7 (0.4, 1.1)	0.097	0.8 (0.5, 1.3)	0.342
**Food Insecurity**				
Secure	Reference		Reference	
Insecure	4.4 (2.2, 9.0)	<0.001	2.4 (1.1, 5.2)	0.028

* Final model: age, sex, country, marital status, multimorbidity and covid impact

# Favourable lifestyle was the outcome variable.

** unemployed, housewife, student, disabled

**Table 5:** Crude and Adjusted Odds Ratios exploring the Association Between Education, Employment, Food Insecurity and Antihypertensive Medication Adherence*#

	Crude Odds Ratio (95% CI)	P-value	Adjusted Odds Ratio (95% CI)	P-value
**Education**				
More than high school	Reference		Reference	
High school	1.2 (0.7, 2.1)	0.554	0.8 (0.4, 1.5)	0.529
Less than high school	1.0 (0.6, 1.9)	0.882	0.8 (0.4, 1.7)	0.519
**Employment**				
Employed/retired	Reference		Reference	
Other **	0.7 (0.4, 1.1)	0.099	0.7 (0.4, 1.3)	0.248
**Food Insecurity**				
Secure	Reference		Reference	
Insecure	3.1 (1.7, 5.6)	<0.001	1.9 (1.0, 3.7)	0.072

* Final model: adjusted for age, sex, country, marital status and healthcare expenses

# Favourable lifestyle was the outcome variable

** unemployed, housewife, student, disabled

## Data Availability

The datasets generated during the current study are available from the corresponding author upon reasonable request.

## Abbreviations

AHA: American Heart Association
ASS-15: Adaptive Significance Scale – 15 item version
BP: Blood pressure
CATCH: Caribbean and south America Team-Based Strategy to Control Hypertension
COVID-19: Corona Virus Diseases 2019
CVDs: Cardiovascular Diseases
DALYs: Disability-adjusted Life Years
FAO: Food and Agriculture Organisation of the United Nations
FIES: Food Insecurity Experience Scale
IMPACTS-MAS: Implementation of Multifaceted Patient-Centred Treatment Strategies for Intensive Blood Pressure Control medication adherence scoring system
LAC: Latin America and the Caribbean
LMICs: Low- and Middle-Income Countries
NCDs: Non-communicable diseases
PAHO: Pan American Health Organisation
SD: Standard Deviations
SDOH: Social determinants of health
USDA: United States Department of Agriculture
WHO: World Health Organization
